# X inactivation in females with X-linked Charcot–Marie–Tooth disease

**DOI:** 10.1016/j.nmd.2012.02.009

**Published:** 2012-07

**Authors:** Sinéad M. Murphy, Richard Ovens, James Polke, Carly E. Siskind, Matilde Laurà, Karen Bull, Gita Ramdharry, Henry Houlden, Raymond P.J. Murphy, Michael E. Shy, Mary M. Reilly

**Affiliations:** a MRC Centre for Neuromuscular Diseases, The National Hospital for Neurology and Neurosurgery and Department of Molecular Neuroscience, UCL Institute of Neurology, London, UK; b Department of Neurology, Adelaide and Meath Hospitals Incorporating the National Children’s Hospital, Tallaght, Dublin, Ireland; c Neurogenetics Department, The National Hospital for Neurology and Neurosurgery and Department of Molecular Neuroscience, UCL Institute of Neurology, London, UK; d Department of Neurology and Center for Molecular Medicine and Genetics, Wayne State University, Detroit, MI, USA

**Keywords:** Charcot–Marie–Tooth disease, GJB1, Connexin32, X inactivation

## Abstract

X-linked Charcot–Marie–Tooth disease (CMT1X) is the second most common inherited neuropathy, caused by mutations in *gap junction beta-1* (*GJB1*). Males have a uniformly moderately severe phenotype while females have a variable phenotype, suggested to be due to X inactivation. We aimed to assess X inactivation pattern in females with CMT1X and correlate this with phenotype using the CMT examination score to determine whether the X inactivation pattern accounted for the variable phenotype in females with CMT1X. We determined X inactivation pattern in 67 females with CMT1X and 24 controls using the androgen receptor assay. We were able to determine which X chromosome carried the *GJB1* mutation in 30 females. There was no difference in X inactivation pattern between patients and controls. In addition, there was no correlation between X inactivation pattern in blood and phenotype. A possible explanation for these findings is that the X inactivation pattern in Schwann cells rather than in blood may explain the variable phenotype in females with CMT1X.

## 1 Introduction

X-linked Charcot–Marie–Tooth disease (CMT1X), caused by mutations in the *gap junction beta-1* gene (*GJB1*), is the second most frequent cause of CMT [1,2]. Over 300 different mutations have been described in *GJB1* to date, spread throughout the coding region. Several mutations have also been described outside the coding region, in the promoter or untranslated regions of the gene, resulting in a similar phenotype as coding region mutations [3,4]. Males have a relatively uniform phenotype, presenting within the first two decades with difficulty walking, distal weakness and sensory loss. Severity increases with age, such that most men are moderately to severely affected by adulthood; this suggests that all mutations cause a loss of function of the connexin32 protein [5]. However, we previously demonstrated that females carrying a *GJB1* mutation have a variable phenotype; approximately two-thirds have a mild non-progressive phenotype, one-third have a moderately severe phenotype that progresses with age, and a small proportion are asymptomatic [6]. This variable phenotype in females has been suggested to occur as a result of X inactivation [6–8].

X inactivation occurs early in embryonic development whereby either the paternally- or maternally-inherited X chromosome is inactivated in each cell. This ensures equivalent expression of sex chromosome genes in males and females [9]. Once X inactivation has occurred in a cell, all subsequent daughter cells have the same X inactivation pattern. The process is usually random such that roughly equal amounts of cells express each X chromosome. In the general population X inactivation pattern is normally distributed; 10–20% of females have a skewed X inactivation pattern ⩾ 80:20 [10,11]. However, carrying a mutation in some X-linked genes has been shown to affect X inactivation pattern [12–14].

We previously investigated X inactivation pattern in 14 females with *GJB1* mutations and found no difference in X inactivation pattern between patients and controls [6]; however, we could not determine which X chromosome carried the mutation and thus were unable to correlate X inactivation pattern with phenotype. In this study, we determined the X inactivation pattern in a large cohort of females and family members carrying *GJB1* mutations to investigate whether X inactivation pattern correlates with phenotype.

## 2 Materials and methods

This study was approved by the Research Ethics Committee at the National Hospital for Neurology and Neurosurgery and the Wayne State University Human Investigation Committee. All patients gave written informed consent to undergo genetic testing.

Females with CMT1X and, where possible, their affected male relatives were recruited. The severity of neuropathy was assessed using the CMT Neuropathy Score (CMTNS) [15]. A total score of 0–10 indicates mild severity; 11–20 moderate severity and ⩾21 severe neuropathy. Where neurophysiological testing was unavailable the CMT Examination Score (CMTES), a subscore of the CMTNS, was used.

We determined the X inactivation pattern in blood using the androgen receptor assay as we have described previously [6]. In brief, >90% of females have a polymorphic CAG repeat region within the androgen receptor gene on the X chromosome [11]. Two CCGG sites 100 bp upstream of this CAG repeat region are methylated on the inactive X chromosome. A restriction digest was performed in females using a methylation sensitive enzyme HpaII which cleaves the active X chromosomes at the unmethylated CCGG sites, leaving the inactive X chromosomes intact. PCR was then performed to amplify this region on the inactive X chromosomes. Size analysis of the PCR products was then performed to determine the ratio of one allele to the other. The terms short and long allele are used (referring to the size of the CAG repeat) to distinguish between one allele and the other.

The ratio of one allele to the other was plotted against the proportion of individuals (patients and controls) to compare X inactivation in patients and controls.

In order to determine whether X inactivation pattern varied by age, we grouped patients into three groups: <30 years, 31–60 years and >60 years. ANOVA was used to compare X inactivation pattern between the three groups.

In order to determine which X chromosome was carrying the *GJB1* mutation, and hence the proportion of mutant allele that was active, we determined the common allele carried by affected family members by using the number of CAG repeats on each allele.

The proportion of mutant allele that was active was plotted against the CMTES and Pearson correlation coefficient calculated to determine whether there was any relationship between X inactivation pattern in blood and phenotype.

## 3 Results

**Fig. 1:**
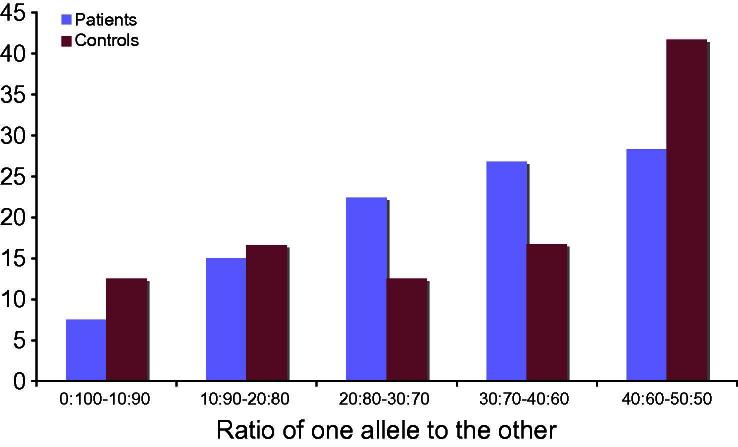
Bar chart demonstrating the ratio of one allele to the other in females with *GJB1* mutations and controls, e.g., 15% of patients and 16.6% of controls had a ratio of one allele to the other of between 10:90 and 20:80.

X inactivation pattern was determined in 67 females with CMT1X (mean age 46 years, range 18–78), 18 affected male relatives and 24 female controls (mean age 51 years, range 23–70). The proportion of long allele active ranged from 3.56% to 97.32% in patients and 2.07% to 84.31% in controls. There was no difference in X inactivation pattern between females with a *GJB1* mutation and controls (Fig. 1).

There was no significant difference in X inactivation pattern when patients were grouped by age (*p* = 0.69).

**Fig. 2:**
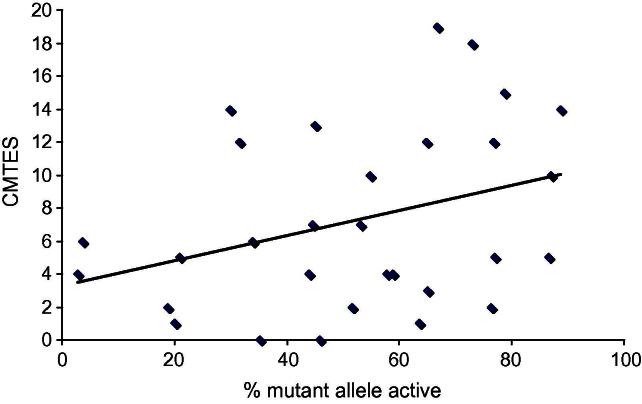
Graph demonstrates no significant correlation (*r* = 0.33, *p* = 0.07) between the percentage mutant allele which is active in blood and the CMT Examination Score (CMTES) (*n* = 30).

**Fig. 3:**
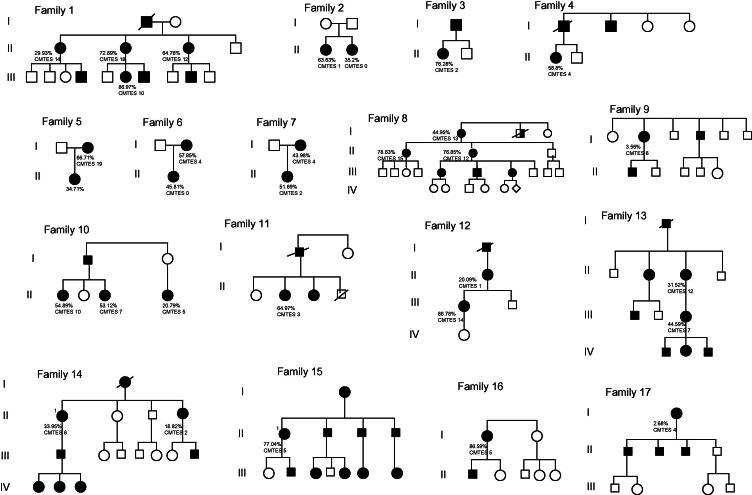
Pedigrees of families demonstrating percentage of mutant allele that is active and CMTES.

We were able to determine which allele carried the *GJB1* mutation and had CMTES data for 30 females (Table 1). When CMTES was plotted against the percentage of mutant allele that was active, there was no association between the two (*r* = 0.33, *p* = 0.07) (Fig. 2). In addition, within and between individual families, the percentage of mutant allele that was active did not correlate with CMT severity (Fig. 3).

## 4 Discussion

The phenotypic difference between males and females with CMT1X is well recognised [8,16]. This has been suggested to occur due to the presence of a non-mutated X chromosome in females [16]. We previously demonstrated that males with CMT1X have a relatively uniform phenotype [5] while females with CMT1X have variable phenotypes, ranging from asymptomatic to as severely affected as males [6]. This variable phenotype in females has been postulated to occur due to X inactivation.

Carrying a mutation in some X-linked genes has been demonstrated to affect X inactivation pattern: in X-linked adrenoleukodystrophy, there is skewing in favour of the mutant X chromosome [14,17], while mutations in dystonia-deafness-peptide 1 cause skewing in favour of the normal X chromosome [12]. In addition, the pattern of X inactivation has been shown to correlate with phenotype in some X-linked diseases: two sisters were described with fragile X-associated tremor ataxia syndrome (FXTAS) in whom the severity of disease correlated with the pattern of X inactivation [18]. However, a correlation between phenotype and X inactivation pattern has not been demonstrated in other X-linked diseases such as Rett syndrome [19,20], haemophilia [21] or Duchenne’s muscular dystrophy [22]. Only one study has investigated X inactivation in CMT1X: Lin et al. investigated X inactivation pattern in blood in the asymptomatic mutation-carrying mother of a severely affected girl with CMT1X and demonstrated that the X inactivation pattern was skewed in favour of the normal allele in the mother; however, they were unable to determine the X inactivation pattern in the daughter due to the methodology used [23].

We previously demonstrated in a small cohort that there was no difference in X inactivation pattern between females with CMT1X and controls [6]; this current study confirms the normal distribution of X inactivation pattern in females with a *GJB1* mutation. In addition, this study demonstrates that there is no correlation between X inactivation pattern in blood and phenotype. However, given that the phenotype in CMT1X is thought to be related to a dosage effect [5], it may be that X inactivation pattern within Schwann cells partly explains the variable phenotype in females. Several studies have shown that the X inactivation pattern in different tissues does not correlate well [24,25]. Comparison of X inactivation pattern has not been performed between blood and peripheral nerve; however, X inactivation pattern between blood (mesodermal origin) and brain tissue (ectodermal origin) can vary considerably in individuals [24]. Thus, X inactivation pattern in myelinating Schwann cells (ectodermal origin) may also be different from that in blood. X inactivation pattern was investigated in blood and tissue from five discrete areas of the liver in a female manifesting ornithine transcarbamylase deficiency. The authors found that X inactivation pattern varied between different areas of liver and the pattern in blood differed from that in liver. However, enzyme activity in each liver sample correlated with X inactivation pattern within that sample [26]. This demonstrates the patchy nature of X inactivation even within a single tissue but supports the hypothesis that X inactivation pattern in the tissue of interest may explain the phenotype in X-linked diseases. Also supporting this hypothesis, in teased nerve fibres of heterozygous *GJB1*+/− mice, Scherer et al. demonstrated examples of apposed paranodes where one paranode stained for connexin32 while the apposed paranode did not, indicating that *GJB1* was subject to X inactivation in mice and that X inactivation was patchy within peripheral nerve tissue [7].

Although differential X inactivation pattern in Schwann cells may be one possible explanation for the variable phenotype in females with *GJB1* mutations, we cannot discount other genetic or environmental factors that may impact on phenotype expression.

In conclusion, females with *GJB1* mutations have a normal distribution of X inactivation pattern and X inactivation pattern in blood does not correlate with phenotype. However, we hypothesise that the X inactivation pattern in the myelinating Schwann cells of peripheral nerves may account, at least in part, for the variable phenotype in females with CMT1X.

## Figures and Tables

**Table 1:** Data for 30 females demonstrating percentage of mutant-carrying allele that is active and CMTES.

% Mutant allele active	CMTES
2.68	4
3.56	6
18.82	2
20.09	1
20.79	5
29.93	14
31.52	12
33.95	6
35.20	0
43.98	4
44.59	7
44.95	13
45.81	0
51.69	2
53.12	7
54.89	10
57.85	4
58.83	4
63.63	1
64.76	12
64.97	3
66.71	19
72.89	18
76.28	2
76.85	12
77.04	5
78.63	15
86.59	5
86.97	10
88.78	14

CMTES = CMT examination score (max score 28).
